# The Effects of Hyperbaric Oxygenation on Oxidative Stress, Inflammation and Angiogenesis

**DOI:** 10.3390/biom11081210

**Published:** 2021-08-14

**Authors:** Silke D. De Wolde, Rick H. Hulskes, Robert P. Weenink, Markus W. Hollmann, Robert A. Van Hulst

**Affiliations:** 1 Department of Anesthesiology, Amsterdam University Medical Centers, Location AMC, 1105 AZ Amsterdam, The Netherlands; r.h.hulskes@amsterdamumc.nl (R.H.H.); r.p.weenink@amsterdamumc.nl (R.P.W.); m.w.hollmann@amsterdamumc.nl (M.W.H.); r.a.vanhulst@amsterdamumc.nl (R.A.V.H.); 2 Department of Hyperbaric Medicine, Amsterdam University Medical Centers, Location AMC, 1105 AZ Amsterdam, The Netherlands; 3 Department of Surgery, Amsterdam University Medical Centers, Location AMC, 1105 AZ Amsterdam, The Netherlands

**Keywords:** hyperbaric oxygen therapy, hyperbaric oxygenation, oxidative stress, inflammation, angiogenesis, neovascularization

## Abstract

Hyperbaric oxygen therapy (HBOT) is commonly used as treatment in several diseases, such as non-healing chronic wounds, late radiation injuries and carbon monoxide poisoning. Ongoing research into HBOT has shown that preconditioning for surgery is a potential new treatment application, which may reduce complication rates and hospital stay. In this review, the effect of HBOT on oxidative stress, inflammation and angiogenesis is investigated to better understand the potential mechanisms underlying preconditioning for surgery using HBOT. A systematic search was conducted to retrieve studies measuring markers of oxidative stress, inflammation, or angiogenesis in humans. Analysis of the included studies showed that HBOT-induced oxidative stress reduces the concentrations of pro-inflammatory acute phase proteins, interleukins and cytokines and increases growth factors and other pro-angiogenesis cytokines. Several articles only noted this surge after the first HBOT session or for a short duration after each session. The anti-inflammatory status following HBOT may be mediated by hyperoxia interfering with NF-κB and IκBα. Further research into the effect of HBOT on inflammation and angiogenesis is needed to determine the implications of these findings for clinical practice.

## 1. Introduction

Since the adjunctive use of hyperbaric oxygen therapy (HBOT) was first described in 1879 [1], it has been further explored and is nowadays a widely accepted treatment in several diseases, such as delayed radiation injury, diabetic foot ulcers, carbon monoxide poisoning, decompression sickness and arterial gas embolism [2]. The Undersea and Hyperbaric Medical Society (UHMS) describes HBOT as an intervention whereby patients breathe near 100% oxygen while being pressurized to at least 1.4 atmosphere absolute (ATA) in a hyperbaric chamber [1]. Currently, the UHMS has accepted 14 indications for HBOT [3], yet new applications of HBOT have been described, including preconditioning for surgery [4,5,6,7].

Several cohort studies and randomized controlled trials, executed in different surgical procedures (e.g., abdominoplasty and pancreaticoduodenectomy), reported lower postoperative complication rates and a reduced length of stay on the intensive care unit after preoperative HBOT [4,5,6,7]. As the occurrence of postoperative complications is associated with worse short-term and long-term outcomes [8], a decrease in psychosocial well-being [9] and higher healthcare costs [10], HBOT may prevent those adverse effects of surgery.

To realize this perioperative protective effect, HBOT must be able to prevent infection and increase wound healing. It is likely that oxidative stress, which has been confirmed to be the main effect of HBOT [11], plays an activating role in the mechanisms underlying the therapeutic pathway of preconditioning for surgery with HBOT. An increase in reactive oxygen species (ROS) levels is associated with enhanced pathogen clearance [12]. Furthermore, ROS induce the synthesis of several growth factors, such as vascular endothelial growth factor (VEGF), placental growth factor (PGF) and angiopoietin (Ang) 1 and 2 and recruit stem cells from the bone marrow, which are responsible for neovascularization [13]. However, a frequently mentioned argument against the use of HBOT revolves around the induction of oxidative stress as well, since higher levels of ROS and reactive nitrogen species (RNS) may lead to oxidative and nitrosative damage, mitochondrial aging, genotoxicity and maintenance of (chronic) inflammation [14,15,16].

The aim of this review is to gain more insight into the mechanisms of HBOT by assessing its effect on oxidative stress, inflammation and angiogenesis markers in humans. More insight into these effects of HBOT will predict and underpin the outcome of innovative uses of HBOT and balance its benefits against potential damage. No systematic overview of research into these parameters in human beings has yet been published.

## 2. Methods

A search of the literature was performed in MEDLINE and EMBASE on 2 November 2020. Key terms used in the search were ‘hyperbaric oxygen’ and ‘oxidative stress’, ‘inflammation’, or ‘wound healing’. The results were not restricted as no filters were applied. The detailed literature search can be found in Appendix A (see Table A1 and Table A2).

All studies found were screened on title and abstract by one reviewer (S.D.D.W.), who excluded those studies that met any of the following criteria: (1) absence of abstract, (2) congress abstract, errata or guideline, (3) case report (defined as five or less patients), (4) narrative review, (5) animal research, (6) no treatment with HBOT, or (7) one of the following outcome measures: cure, complication rate, or a disease-specific outcome parameter. The same reviewer assessed the full-text of the remaining studies. The following inclusion criteria were applied: (1) measurement of at least one marker of oxidative stress, inflammation, or angiogenesis before and after HBOT, (2) study in humans (or human material) and (3) English full-text available. EndNote X9 was used to keep track of the screening process.

The included studies were divided into an “in vivo” and “in vitro” group. In vivo studies were performed in a clinical setting in which all subjects were at least pressurized once, whereas in vitro studies obtained human material what was subsequently exposed to HBOT. Information on first author, publication year, investigated parameters and patient (in vivo)/sample (in vitro) characteristics and results (solely of the parameters of interest) were extracted. Outcomes of statistical tests with a *p*-value < 0.05 were considered significant. All information was extracted by hand and documented in Microsoft Excel (v16.0).

## 3. Results

### 3.1. Eligible Studies

**Figure 1:**
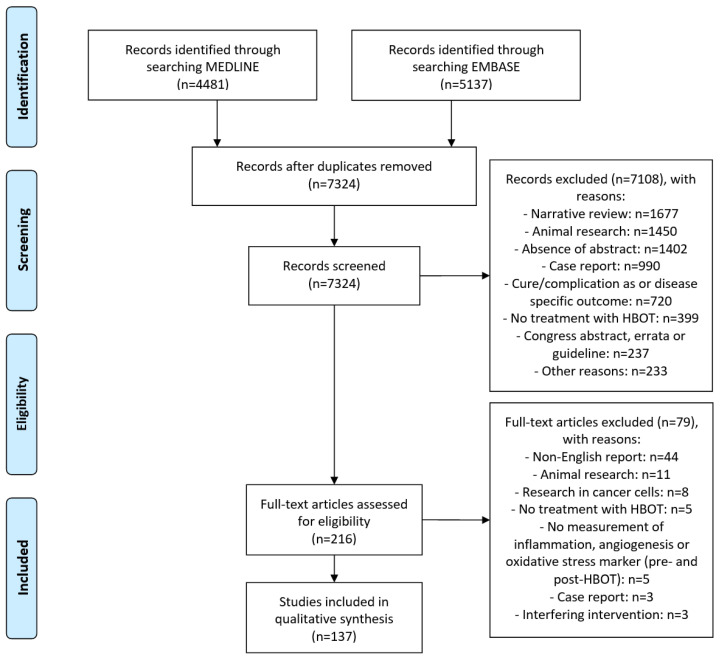
PRISMA Flow Diagram of the selection progress.

The search retrieved 9618 records. After removing duplicates and screening of title and abstract, 216 studies were screened full-text. Finally, 137 articles were included in this review (see Figure 1). Most of the included articles were clinical studies (*n* = 98) and performed in patients with diabetes mellitus and/or non-healing chronic wounds (*n* = 27). Furthermore, 27 articles describing the effect of HBOT in healthy volunteers (including divers) were found. Sixteen included studies reported on other biomarkers than described in Table 1, Table 2 and Table 3 (data not shown) [17,18,19,20,21,22,23,24,25,26,27,28,29,30,31,32].

### 3.2. Oxidative Stress

In total, 74 articles reporting on the effect of HBOT on oxidative stress were found. Subjects mainly received one session of HBOT in a hyperbaric chamber pressurized to 2–2.5 ATA (203–253 kPa), yet in seven studies a wet exposure to hyperbaric oxygen (i.e., a dive) to up to 6 ATA (608 kPa) was employed. Nearly 40% (*n* = 21) of the clinical studies were conducted in healthy volunteers (see Table A3). Catalase, glutathione peroxidase (GPx), malondialdehyde (MDA), nitric oxide synthase (NOS), ROS, RNS, superoxide dismutase (SOD) and thiobarbituric acid reactive substances (TBARS) were the most frequent markers of interest (see Table 1).

A clear stimulating effect of HBOT on ROS (see Table 1) was found. Nonetheless, two out of the three studies assessing hydrogen peroxide described lower concentrations after HBOT [33,42] (see Table A3). NOS and RNS concentrations seem to increase after HBOT as well, although this effect was less pronounced, which can be explained by a repeatedly reported decrease in exhaled nitric oxygen [61,69,70]. Timing of sampling may also play a role, as several articles only noted an increase in inducible NOS or nitrite three hours after the end of an HBOT session [34,49,55].

Not only the presence of NOS, RNS and ROS has been investigated, but also their effects on lipids, proteins, carbohydrates and DNA/RNA (see Table 1). Little research has been done regarding protein and carbohydrate modifications following HBOT, but no effect or a stimulating effect on lipid peroxidation, resulting in MDA and other aldehydes (TBARS), has been reported in various studies. DNA-damaging effects of HBOT were not demonstrated employing the most commonly used DNA-lesion-marker 8-hydroxydeoxyguanosine [146].

Concerning the concentrations of anti-oxidative enzymes that protect against the potentially harmful effects of oxidative stress, such as catalase, SOD and GPx, conflicting results were found (see Table 1). In general, no effect or an indication for an increasing effect of HBOT on the enzyme activity of those antioxidants has been demonstrated. HBOT may have a uniform effect on SOD and catalase, as most of the studies reported increased, decreased, or stable SOD and catalase levels and, thus, no differences in effect of HBOT between these two enzymes [76,80,81,83,85,94,97,100]. However, a difference between SOD and/or catalase concentrations in respectively plasma and erythrocytes has been reported [55,62,63]. Benedetti et al. [80] and Dennog et al. [94] describe no effect of HBOT on the free radical trapping anti-oxidants with an exogenous origin, such as vitamin A, vitamin C and vitamin E [149].

### 3.3. Inflammation

Of the 140 studies included, 58 articles describing inflammatory markers were identified. Most of the research included at least three HBOT-sessions, yet study protocols consisting of 20–40 sessions were common, in particular in articles reporting acute-phase proteins (see Table A4). Popular variables of interest were interleukins (IL) (*n* = 31), acute-phase proteins (*n* = 26) and tumor necrosis factor-α (TNF-α) (*n* = 25) (see Table 2).

Concerning acute phase proteins, a decreasing effect of HBOT on (high-sensitivity) C-reactive protein ((hs-)CRP) was found as 75% (*n* = 12) of the studies investigating (hs-) CRP reported lower concentrations post-HBOT. Strikingly, HBOT may have a stimulating impact on granulocyte-colony stimulating factor and an inhibiting effect on insulin-like growth factor-1, both reflecting a pro-inflammatory state [150] (see Table 2).

No impact of HBOT on most interleukin concentrations (IL-2, IL-3, IL-5, IL-7, IL-9, IL-12p70, IL-13, IL-15, IL-17, IL-18 and IL-22) has been demonstrated, although Hao et al. [111] reported a decrease in IL-12p40 levels (see Table A4). Concerning the proinflammatory interleukins, a potentially inhibiting effect of HBOT on IL-1β, IL-6 and IL-8 was found, whereas Dhadmodharan et al. [45] suggested an increase in IL-1α levels. On the other hand, a rise in the anti-inflammatory IL-1Ra was reported, alongside a possible inhibiting effect of HBOT on IL-10 and no effect on IL-4. Both results support an anti-inflammatory state (see Table 2) [151].

In line with the outcomes regarding (hs-)CRP and interleukins, an anti-inflammatory effect of HBOT was also shown by decreasing levels of the pro-inflammatory cytokines interferon-γ (IFN-γ), nuclear factor kappa B (NF-κB) and TNF-α (see Table 2). However, HBOT may have an initial pro-inflammatory effect, as some studies described an increase in TNF-α during or shortly after HBOT [87,127,134].

### 3.4. Angiogenesis

Concerning the angiogenesis research, 34 studies were found in addition to the earlier mentioned studies reporting on interleukins, interferons, insulin-like growth factor 1 (IGF-1), NF- κB and TNF-α. Most of the articles described angiogenesis-inducing cytokines or growth factors and were performed in clinical setting (*n* = 20). However, five out of seven studies on downstream effectors of angiogenesis were conducted in vitro (see Table A5). Epidermal growth factor (EGF), extracellular signal-regulated kinase (ERK), (basic) fibroblast growth factor, tumor growth factor-β (TGF-β), VEGF, IFN-γ, IL-6, IL-8, NF-κB and TNF-α (see Table 2 and Table 3) were the only angiogenesis markers reported in at least five articles.

HBOT most likely has a stimulating effect on various growth factors involved in angiogenesis (i.e., EGF, hematopoietic growth factor, keratinocyte growth factor, PGF and VEGF). This effect may only be present shortly after the intervention, since several studies with repeated HBOT sessions described no differences in pre-HBOT values or only a raise after the first session (and not after following sessions) [62,141,147] (see Table A5). Whereas for some angiogenesis-stimulating cytokines, such as stromal cell-derived factor-1α, a similar increasing effect of HBOT was found, no or an inhibiting effect on TGF was seen. HBOT seems not to affect the cytokine receptors (see Table 3).

HBOT decreased matrix metalloproteinases (MMPs) [52,54,72]. According to Niu et al. [52,72], the effect on MMPs is delayed and only manifests after two or three HBOT sessions. Hypoxia-inducible factor-1α (HIF-1α) and NF-κB were inhibited by HBOT (see Table 2 and Table 3), although Anguinano-Hernandez et al. [125] described an increase in NF-κB in the cytosol.

As HBOT causes an increase in angiogenesis-promoting growth factors and cytokines, one would also expect a stimulating effect on the downstream effectors of blood vessel formation. However, inconsistent outcomes were reported (see Table 3). The phosphatidylinositol-3 kinase (PI3K)/AKT pathway was upregulated and the ERK and p38 mitogen-activated protein kinase (p38 MAPK) pathways were downregulated. Therefore, HBOT effects on downstream effectors of blood vessel formation seem to differ depending on the intracellular effector route.

## 4. Discussion

This review is the first to systematically summarize the effect of HBOT on oxidative stress, inflammation and angiogenesis markers in human beings. HBOT increases the levels of oxygen radicals, which induce oxidative stress. An anti-inflammatory action of HBOT was demonstrated by decreasing concentrations of several pro-inflammatory markers. Furthermore, HBOT seems to stimulate the release of angiogenesis-promoting cytokines, including growth factors.

In the light of previous research, reporting a link between oxidative stress and a pro-inflammatory state [152,153,154], it is remarkable that HBOT leads to a more anti-inflammatory state. However, these findings do correspond with studies into the effects of HBOT using thermal imaging, in which a decrease in wound temperature was found [155,156]. This temperature reduction could indicate a local decline in inflammation. This anti-inflammatory effect is likely mediated by the inhibition of NF-κB, a transcription factor for pro-inflammatory genes [157,158,159]. A direct anti-inflammatory action of HBOT seems less probable, since no differences in the concentrations of anti-inflammatory markers (except IL-1Ra) were noted. Although beyond the scope of this review, Yu et al. [160] have shown in an animal model that HBOT decreases the NF-κB concentrations by higher release of IκBα, which is an inhibitor of NF-κB and degrades under hypoxic circumstances [161]. An increase in IκBα along with a decrease in NF-κB after HBOT was also seen in the only study in the current review reporting on IκBα [52]. Therefore, hyperoxia generated during HBOT may stimulates the preservation of IκBα and thereby inhibits NF-κB release, resulting in less gene transcription of pro-inflammatory cytokines and, thus, an anti-inflammatory state despite oxidative stress.

NF-κB is not only a crucial transcription factor in inflammation, but also plays a role, together with HIF-1α, in the induction of angiogenesis. Growth factors and other angiogenesis-promoting cytokines induce new vessel formation by increased expression of pro-angiogenesis genes, which is mediated by NF-κB or (under hypoxia) HIF-1α [162,163]. Since the current review demonstrates an inhibiting effect of HBOT on both transcription factors and little research, with contradicting outcomes, into the downstream effectors of angiogenesis (i.e., PI3K, Akt, p38 MAPK, ERK) has been done, it is unclear how increased levels of pro-angiogenesis growth factors and cytokines actually induce increased tube formation, as shown by Anguiano-Hernandez et al. [125], Lin et al. [130] and Shyu et al. [40]. Thus, further research into the relation between NF-κB, HBOT and the angiogenesis pathways is needed.

Another striking finding concerning angiogenesis is that several articles reported an increase in growth factors only or particularly after the first HBOT session [40,141,147], while it is common to conduct 20–40 sessions for chronic non-healing wounds or radiation-induced tissue injury (indications strongly relying on the angiogenesis effects of HBOT) [2]. Furthermore, Sureda et al. [62] describe, in the only in vivo study assessing the effect of HBOT on growth factors at several time points during follow-up, an increase in VEGF immediately after each session, yet VEGF levels determined pre-session #5 and #20 were similar to the baseline (pre-session #1) value. Those findings possibly suggest a short pro-angiogenesis effect of HBOT. However, due to a shortage of studies reporting on angiogenesis markers on a daily or weekly basis during a treatment protocol including 20–40 sessions, it remains unclear which markers are involved in this short-term effect of HBOT and whether other factors play a role in this angiogenesis process.

The aim of this review was to gather a comprehensive overview of the effects of HBOT on oxidative stress, inflammation and angiogenesis. We must conclude that existing research does not allow for a complete understanding of the physiology underlying new promising treatment modalities for HBOT, such as preconditioning for surgery. Due to the heterogeneity of included patient populations and the inclusion of studies in healthy volunteers, it is difficult to extrapolate findings to the surgical patient in general. Furthermore, this review did not focus on clinical outcomes related to inflammation, angiogenesis and oxidative stress, making it impossible to determine the implications of the described findings in practice. In conclusion, hyperoxia and oxidative stress induced by HBOT affect inflammation and angiogenesis markers, but whether hyperoxia and oxidative stress induce a clinically relevant decrease in inflammation and increase in angiogenesis remains unclear and needs to be further investigated before innovative interventions can be widely applied.

## Figures and Tables

**Table 1:** The effect of HBOT on oxidative stress markers.

Main Aspect	Associated Markers	Stimulating Effect	No Effect	Inhibiting Effect
Causers of oxidative stress	Reactive oxygen species (including superoxide-ion and hydrogen peroxide)	[33,34,35,36,37,38,39,40,41]	[33,37,38,42,43]	[33,42,43,44]
	Nitric oxide synthase (NOS) (including endothelial NOS and inducible NOS)	[34,45,46,47,48]	[49,50,51]	[52,53,54]
	Reactive nitrogen species (including nitric oxygen, nitrite and nitrate)	[33,34,45,49,55,56,57,58,59,60]	[33,46,61,62,63,64,65,66,67,68]	[53,61,67,68,69,70,71,72,73]
	Hydrobenzoates	[74,75]	[74]	
	Free fatty acid			[53]
	Myeloperoxidase			[34,62]
Lipid peroxidation	Isoprostanes	[76,77]	[78,79]	
	Isofurans		[78]	
	Malondialdehyde	[56,77,80,81,82]	[14,34,49,55,76,83]	[62,84]
	Thiobarbituric acid reactive substances	[63,74,85,86,87]	[33,63,85,88,89,90]	
	Lipid hydroperoxides	[36]	[91]	[92]
	Oxidized low-density lipoprotein		[82]	
Protein peroxidation	Nitrotyrosine	[49]	[64]	
	Advanced oxidation protein products			[77]
Carbohydrate peroxidation	Protein carbonyls	[93]		
	Carbonyl group	[56]		
	Protein carbonyl derivates		[55]	
	Plasma carbonyl proteins		[83]	[82]
DNA/RNA damage	8-hydroxydeoxyguanosine		[83]	[82]
	Tail moment	[38,94,95,96]	[76,97,98]	
	Sister chromatid exchange	[14]		
Gene expression	Nuclear factor erythroid 2- related factor 2	[45]		
Other residues of oxidative stress	Reactive oxygen metabolites	[80]		
	Intracellular calcium concentration			[43]
Antioxidants	Total antioxidant capacity		[91,93,94,99]	
	Catalase	[33,34,43,45,55,62,81,100]	[43,55,62,63,76,83,89,94,97]	[63,80,85]
	Superoxide dismutase	[55,56,81,84,100,101]	[14,33,55,62,63,76,83,89,94,97,102]	[80,85,86]
	Glutathione	[92]	[76,80,83,94]	
	Glutathione disulfide		[76,101]	
	Glutathione reductase		[55,62]	
	Glutathione peroxidase	[34,63,82,85]	[14,55,62,63,76,80,83,89,94,100]	[63]
	Thiols		[88]	[93]
	Vitamin A		[80,94]	
	Vitamin C		[94]	
	Vitamin E		[80,94]	
	Uric acid		[91,102]	[103]
	Heme oxygenase-1	[45,95,96,97]		
	NAD(P)H dehydrogenase [quinone] 1	[45]		

**Table 2:** The effect of HBOT on inflammation markers.

Main Aspect	Associated Markers	Stimulating Effect	No Effect	Inhibiting Effect
Acute-phase proteins	(high-sensitivity) C-reactive protein		[88,101,104,105]	[45,53,58,59,60,84,101,103,106,107,108,109]
	Granulocyte-colony stimulating factor	[110]	[110,111,112]	
	Ferritin		[96]	
	Insulin-like growth factor-1	[113]	[67,114,115]	[114,115]
	Albumin	[116]	[102,117]	
Interleukins (IL)	IL-1α	[45]	[111,112]	
	IL-1β		[35,87,111,112,118,119]	[71,72,73,120,121,122]
	IL-1Ra	[111]	[123]	
	IL-1		[4]	[124]
	IL-4		[111,112,118,125,126]	
	IL-6	[62,123,125]	[87,104,105,111,112,118,123,127,128]	[4,35,122,124,129,130,131,132]
	IL-8	[45]	[4,67,105,110,111,112,128]	[46,105,127]
	IL-10	[45]	[106,107,113,114,120,127,128]	[4,105]
Interferons (IFN)	IFN-α		[111,112]	
	IFN-γ	[118]	[111,112,125,126]	[45,133]
Cytokines	Tumor necrosis factor-α	[87,127,134]	[4,45,111,112,128,135,136]	[35,84,105,119,120,121,123,124,129,131,132,133,137,138]
	Nuclear factor kappa B	[139]	[125]	[52,53,125,132,137,140]
Others	Erythrocyte sedimentation rate	[107]		[106,108]

**Table 3:** The effect of HBOT on angiogenesis markers.

Main Aspect	Associated Markers	Stimulating Effect	No Effect	Inhibiting Effect
Growth factors/ cytokines	Vascular endothelial growth factor	[45,62,84,125,130,138,141,142]	[50,51,113,120,127,137,143,144,145,146]	[132]
	(basic) Fibroblast growth factor	[45,141]	[102,111,147,148]	[141,143]
	Platelet-derived growth factor		[45,111]	[111]
	Insulin-like growth factor-binding protein	[125]	[67,125]	[67]
	Epidermal growth factor	[45]	[50,111,135,136]	
	Insulin-like growth factor-2		[67]	
	Hematopoietic growth factor	[130,141]	[136]	
	Keratinocyte growth factor	[141]		
	Placental growth factor	[40,141]		[141]
	Tumor growth factor-α		[111]	
	Tumor growth factor-β		[112,147,148]	[115,136]
	Angiopoietin	[84]	[144]	
	Erythropoietin			[145]
	Granulocyte-macrophage colony-stimulating factor		[111,112]	
	Stromal cell-derived factor-1α	[130]		
Cytokine receptors	Tie-2		[144]	
	Erythropoietin-receptor		[37]	
Proteases	Matrix metalloproteinase-3			[52,54,72]
	Matrix metalloproteinase-9			[52]
	Matrix metalloproteinase-13			[52]
Transcription factors	Hypoxia-inducible factor-1α		[125]	[47,130,132]
Downstream effectors	Phosphatidylinositol-3 kinase (PI3K)	[48]		
	AKT	[48]		[53]
	p38 mitogen-activated protein kinase (p38 MAPK)			[44,52,71,72]
	Extracellular signal-regulated kinase (ERK)	[142]	[44]	[52,53,71]

## Appendix A

**Table A1:** Search strategy used in PubMed (MEDLINE).

Key Terms	Mesh-Terms	Title/Abstract-Terms
Hyperbaric oxygen	hyperbaric oxygenation[MeSH]	hyperbaric oxygen[tiab] OR hyperbaric oxygenation[tiab] OR hyperbaric oxygen therapy[tiab] OR hyperbaric oxygen therapies[tiab] OR HBO[tiab] OR HBOT[tiab] OR hyperbaric medicine[tiab]
	**AND**	
Inflammation	inflammation[MeSH] OR inflammation mediators[MeSH] OR autacoids[MeSH] OR chemokines[MeSH] OR synthetic prostaglandins[MeSH] OR interleukin[MeSH] OR infection[MeSH]	inflammation[tiab] OR inflammations[tiab] OR inflammatory[tiab] OR inflammatory respons[tiab] OR inflammation mediators[tiab] OR mediators of inflammation[tiab] OR chemokines[tiab] OR slow reacting substances[tiab] OR chemotactic cytokines[tiab] OR synthetic prostaglandins[tiab] OR intercrines[tiab] OR PG analogs[tiab] OR prostaglandin analogues[tiab] OR prostaglandin analogs[tiab] OR interleukin[tiab] OR infection[tiab] OR infection and infestation[tiab] OR infections and infestations[tiab] OR infestation and infection[tiab] OR infestations and infections[tiab]
	**OR**	
Wound healing	wound healing[MeSH] OR re-epithelialization[MeSH] OR angiogenesis modulating agents[MeSH] OR neovascularization, physiologic[MeSH] OR cell proliferation[MeSH]	woundhealing[tiab] OR wound healing[tiab] OR cicatrization[tiab] OR re-epithelialization[tiab] OR wound epithelialization[tiab] OR angiogenesis[tiab] OR angiogenesis modulating agents[tiab] OR vasculogenesis[tiab] OR blood vessel formation[tiab] OR bloodvessel formation[tiab] OR neovascularization[tiab] OR neovascularisation[tiab] OR cell proliferation[tiab] OR endothelial proliferation[tiab] OR vascular proliferation[tiab]
	**OR**	
Oxidative stress	Oxidative stress[MeSH] OR nitrosative stress[MeSH] OR reactive oxygen species[MeSH] OR reactive nitrogen species[MeSH]	oxidative stress[tiab] OR nitrosative stress[tiab] OR reactive oxygen species[tiab] OR reactive nitrogen species[tiab] OR peroxide[tiab] OR peroxides[tiab] OR superoxide[tiab] OR superoxides[tiab] OR hydroxy radical[tiab] OR hydroxy radicals[tiab] OR singlet oxygen[tiab] OR alpha-oxygen[tiab] OR nitric oxide[tiab] OR peroxynitrite[tiab] OR nitrogen dioxide[tiab] OR oxidant stress[tiab] OR reactive oxygen metabolite[tiab] OR reactive oxygen metabolites[tiab] OR reactive nitrogen metabolite[tiab] OR reactive nitrogen metabolites[tiab]

**Table A2:** Search strategy used in Ovid (EMBASE).

Key-Terms.	Emtree-Terms	Title/Abstract/Author Keywords-Terms
Hyperbaric oxygen	hyperbaric oxygen/ OR hyperbaric oxygen therapy/	hyperbaric oxygen therapy.ti,ab,kw OR hyperbaric oxygen therapies.ti,ab,kw OR HBOT.ti,ab,kw OR hyperbaric oxygenation.ti,ab,kw OR hyperbaric oxygen.ti,ab,kw OR HBO.ti,ab,kw OR hyperbaric medicine.ti,ab,kw
	**AND**	
Inflammation	Inflammation/ OR inflammation autocoid/ OR chemokine/ OR chronic inflammation/ OR inflammation/ OR cytokine/ OR infection/	inflammation.ti,ab,kw OR inflammations.ti,ab,kw OR inflammatory.ti,ab,kw OR inflammatory respons.ti,ab,kw OR inflammation mediators.ti,ab,kw OR mediators of inflammation.ti,ab,kw OR chemokines.ti,ab,kw OR slow reacting substances.ti,ab,kw OR chemotactic cytokines.ti,ab,kw OR synthetic prostaglandins.ti,ab,kw OR intercrines.ti,ab,kw OR PG analogs.ti,ab,kw OR prostaglandin analogues.ti,ab,kw OR prostaglandin analogs.ti,ab,kw OR interleukin.ti,ab,kw OR infection.ti,ab,kw OR (infection and infestation).ti,ab,kw OR (infections and infestations).ti,ab,kw OR (infestation and infection).ti,ab,kw OR (infestations and infections).ti,ab,kw
	**OR**	
Wound healing	wound healing/ OR epithelialization/ OR angiogenesis/ OR cell proliferation/	woundhealing.ti,ab,kw OR wound healing.ti,ab,kw OR cicatrization.ti,ab,kw OR re-epithelialization.ti,ab,kw OR wound epithelialization.ti,ab,kw OR angiogenesis.ti,ab,kw OR neovascularization.ti,ab,kw OR neovascularisation.ti,ab,kw OR (angiogenesis modulating agents).ti,ab,kw OR vasculogenesis.ti,ab,kw OR blood vessel formation.ti,ab,kw OR bloodvessel formation.ti,ab,kw OR cell proliferation.ti,ab,kw OR endothelial proliferation.ti,ab,kw OR vascular proliferation.ti,ab,kw
	**OR**	
Oxidative stress	oxidative stress/ OR nitrosative stress/ OR reactive oxygen metabolite/ OR reactive nitrogen species/	oxidative stress.ti,ab,kw OR nitrosative stress.ti,ab,kw OR reactive oxygen species.ti,ab,kw OR reactive nitrogen species.ti,ab,kw OR peroxide.ti,ab,kw OR peroxides.ti,ab,kw OR superoxide.ti,ab,kw OR superoxides.ti,ab,kw OR hydroxy radical.ti,ab,kw OR hydroxy radicals.ti,ab,kw OR singlet oxygen.ti,ab,kw OR alpha-oxygen.ti,ab,kw OR nitric oxide.ti,ab,kw OR peroxynitrite.ti,ab,kw OR nitrogen dioxide.ti,ab,kw OR oxidant stress.ti,ab,kw OR reactive oxygen metabolite.ti,ab,kw OR reactive oxygen metabolites.ti,ab,kw OR reactive nitrogen metabolite.ti,ab,kw OR reactive nitrogen metabolites.ti,ab,kw

**Table A3:** Retrieved results on oxidative stress markers in detail.

Study	Year	Design	Subjects	Methods			Type oxidative Stress Marker	Outcome	Remarks
				Amount of Sessions	Maximum Pressure (ATA) ^a^	Moment of Sample Taking ^b^			
Ansari et al. [100]	1986	In vivo	18 patients; multiple sclerosis	20	2	Within 30 min post-HBOT	Catalase, GPx, SOD	Catalase: increase SOD: increase *GPx: no significant differences were found*	No effect of HBOT was seen in the group breathing chamber air at 2 ATA
Bearden et al. [86]	1999	In vivo	10 divers	1	1.4–1.5 (dive)	Within 15 min post-dive	SOD, TBARS	SOD: decrease TBARS: increase	
Benedetti et al. [80]	2004	In vivo	12 patients; several pathological conditions	15	2.5	Immediately post-session #1 and #15	Catalase, glutathione, GPx, MDA, reactive oxygen metabolites, retinol (vitamin A), SOD, α-tocopherol (vitamin E)	Catalase: decrease MDA: increase Reactive oxygen metabolites: increase SOD: decrease *Glutathione, GPx, retinol (vitamin A), a-tocopherol (vitamin E): no significant differences were found*	Reported outcomes are based on a comparison of the pre-session values of sessions #1 and #15. No significant differences in post-session values were found
Bosco et al. [35]	2018	In vivo	23 patients; unilateral femoral head necrosis	60	2.5	Post-session #15, #30, and #60 and pre-session #31	ROS	Increase at post-session #15, post-session #30 and pre-session #31	
Boykin et al. [57]	2007	In vivo	6 patients; chronic non-healing wound	20	2	Post-session #10, post-session #20, 1 week post-HBOT, and 4 weeks post-HBOT	Nitric oxygen	Increase at 1 week post-HBOT and 4 weeks post-HBOT	
Burgos et al. [91]	2016	In vivo	12 young soccer players	15	2	Pre- and post-session #5, #10, and #15	Antioxidant capacity, lipid hydroperoxides, uric acid	*No significant differences were found*	Results are possible influenced by exercising during HBOT
Chang et al. [102]	2020	In vivo	10 healthy male volunteers	1	2.8	30 min, 2 days, and 1 week post-HBOT	Uric acid	*No significant differences were found*	
Chen et al. [36]	2011	In vitro	Blood samples of healthy males	1	1.5, 2, and 2.5	?	Lipid peroxides, superoxide-ion	Lipid peroxides: increase Superoxide-ion: increase	
Chen et al. [88]	2018	In vivo	50 patients; acute non-cardioembolic stroke	1	2.5	1 month post-HBOT	TBARs, thiols	*No significant differences were found*	
Chen et al. [67]	2007	In vivo	31 patients with diabetes mellitus type 2 and 29 healthy volunteers	3	2.5	Immediately post-session #1 and post-session #3	Nitric oxygen	Decrease in diabetes mellitus group	
Cheung et al. [37]	2018	In vitro	Umbilical cord blood enriched with CD34-cells	1	2.5	24 h post-HBOT	ROS	Increase in nucleus and mitochondria	No significant differences were found in the cytoplasm
Corcoran et al. [78]	2017	In vivo	12 patients; osteonecrosis secondary to radiation therapy	1	2.4	During HBOT, immediately post-HBOT, and 30 min post-HBOT	Isofurans, isoprostanes	*No significant differences were found*	
Dejmek et al. [164]	2018	In vitro	Human fetal lung fibroblasts	5	3	?	SOD	Increase	
Dennog et al. [94]	1999	In vivo	Healthy volunteers	1	2.5	Immediately and 24 h post-HBOT	Catalase, glutathione, GPx, SOD, tail moment, total antioxidant capacity, vitamin A, vitamin C, vitamin E	Tail moment: increase *Catalase, glutathione, GPx, SOD, total antioxidant capacity, vitamin A, vitamin C, vitamin E: no significant differences were found*	
Dhamodharan et al. [45]	2019	In vivo	37 patients; diabetic foot ulcer	25	2.2	20 days after the first HBOT-session	Catalase, endothelial NOS, heme oxygenase-1, NAD(P)H dehydrogenase [quinone] 1, nitrite, nuclear factor erythroid-2 related factor 2	Catalase: increase Endothelial NOS: increase Heme oxygenase-1: increase NAD(P)H dehydrogenase [quinone] 1: increase Nitrite: increase Nuclear factor erythroid-2 related factor 2: increase	
Dise et al. [92]	1987	In vivo	Adult male volunteers	1	3	Within 60 min post-HBOT and 24 h post-HBOT	glutathione, lipid hydroperoxides	Glutathione: increase Lipid hydroperoxides: decrease	
Dragic et al. [65]	2020	In vivo	64 patients; peripherial arterial disease	10	2.2	Post-session #10	Nitric oxygen	*No significant differences were found*	
Eken et al. [14]	2005	In vivo	15 patients	20	2.5	Immediately post-session #1, #10, and #20	GPx, MDA, sister chromatide exchange, SOD	Sister chromatide exchange: increase *GPx, MAD, SOD: no significant differences were found*	
Ferrer et al. [34]	2007	In vivo	7 male divers and 12 male physically active volunteers	1	5 (dive)/2.2	Immediately and 3 h post-dive/30 min post-HBOT	catalase, GPx, hydrogen peroxide, inducible NOS, MDA, myeloperoxidase, nitrite	Catalase: increase 3 h post-dive GPx: increase Hydrogen peroxide: increase 3 h post-dive and post-HBOT Inducible NOS: increase 3 h post-dive Myeloperoxidase: decrease 3 h post-dive Nitrite: increase 3 h post-dive *MDA: no significant differences were found*	
Gasier et al. [63]	2013	In vivo	12 healthy male divers	3	1.5/2	15 min, 1 h, and 2 h after each session	Catalase, GPx, nitrite, SOD, TBARS	Catalase: decrease in erythrocytes post-HBOT at 2 ATA and 1 h post-HBOT at 1.5 ATA GPx: increase in erythrocytes post-HBOT at 1.5 ATA and a decrease in erythrocytes post-HBOT at 2 ATA TBARS: increase in erythrocytes 15 min post-HBOT at 1.5 ATA *Nitrite, SOD: no significant differences were found*	No significant differences in catalase, GPX, and TBARS were found in the plasma
Grimberg-Peters et al. [44]	2016	In vitro	Neutrophils from severely injured patients and healthy volunteers	1	2	?	ROS	Decrease	After 3 h stimulation with PMA
Gröger et al. [38]	2009	In vitro	Lymphocytes from combat swimmers, divers, and nondiving volunteers	1	4	Immediately, 1 h, and 2 h post-HBOT	Superoxide-ion, tail moment	Superoxide-ion: increase Tail moment: increase immediately post-HBOT	No increase in superoxide radical was seen in the combat swimmers group, which had high baseline superoxide radical levels. Superoxide radical has been measured only once (at which measurement point is unknown)
Gronow et al. [74]	2005	In vivo	28 divers and 10 volunteers	1	1.7 (dive)/2.8	?	Hydrobenzoates, TBARS	Hydrobenzoates: increase TBARS: increase	No significant differences were found concerning monohydrobenzoates
Gurdol et al. [68]	2010	In vivo	18 patients; diabetic foot ulcers	25/30	2.4	Post session #25/#30	Nitric oxygen	*No significant differences were found*	A decrease in NO levels post-HBOT was seen in the group with <50% wound healing, which had significantly higher baseline NO values
Gürdöl et al. [77]	2008	In vivo	20 patients; type 2 diabetic with foot ulcers	15	2.4	30 min post-session #1 and #15	Advanced oxidation proteins products, isoprostanes, MDA	Advanced oxidation proteins products: decrease at pre-session #15 Isoprostanes: increase post-session #15 MDA: increase post-session #1	
Handy et al. [99]	2005	In vivo	31 patients; non-healing wounds	20	2.2	Immediately post-session #1 and #20	Total antioxidant capacity	*No significant differences were found*	
Kähler et al. [75]	2013	In vivo	118 volunteers	1	2.4/2.8	?	Dihydroxylated benzoate	Increase	Administration of 100% oxygen significantly increased the dihydroxylated benzoate levels, yet pressurization had no extra effect.
Karadurmus et al. [103]	2010	In vivo	28 patients; diabetic foot ulcers	30	2.4	Post-session #10, #20, and #30	Uric acid	Decrease	
Kendall et al. [46]	2012	In vitro	Human umbilical vein endothelial cells	1	2.4	Immediately, 5 h, and 22.5 h post-HBOT	Endothelial NOS, nitrate + nitrite, nitric oxygen	Endothelial NOS: increase *Nitrate + nitrite, nitric oxygen: no significant differences were found*	
Kendall et al. [42]	2013	In vitro	Human umbilical vein endothelial cells	1	2.4	?	Hydrogen peroxide, superoxide-ion	Hydrogen peroxide: decrease *Superoxide-ion: no significant differences were found*	
Kot et al. [93]	2003	In vivo	96 healthy volunteers	1	2.8	Immediately post-HBOT	Protein carbonyls, total antioxidant status, total thiol	Protein carbonyls: increase Total thiol: decrease *Total antioxidant status: no significant differences were found*	
Kozakiewicz et al. [56]	2018	In vivo	42 healthy volunteers	1	3	?	Carbonyl group, MDA, nitrate/nitrite, SOD	Carbonylgroep: increase MDA: increase Nitrate/nitrite: increase SOD: increase	The baseline values were significantly higher (carbonyl group) and lower (nitrate/nitrite and SOD-1) in the HBOT-group compared to the control group
Lambrechts et al. [64]	2013	In vivo	10 military divers	1	4 (dive)/1.7	1 h post-dive/1 h post-HBOT	Nitrotyrosine, nitric oxygen	*No significant differences were found*	
Li et al. [84]	2017	In vivo	78 patients; chronic diabetic wounds	By average 48	2.4	30 days after the first HBOT-session	MDA, SOD	MDA: decrease SOD: increase	
Li et al. [58]	2018	In vivo	115 patients; coronary artery disease with drug-eluting stents	24	2	?	Nitric oxygen	Increase	
Li et al. [59]	2019	In vivo	115 patients; coronary artery disease with coronary stent implantation	24	2	?	Nitric oxygen	Increase	
Li et al. [60]	2018	In vivo	98 patients; slow coronary flow	24	2	?	Nitric oxygen	Increase	
Lin et al. [39]	2008	In vitro	Detroit 551 normal human dermal fibroblasts	3	2.5	?	ROS	Increase	
Ma et al. [81]	2013	In vivo	36 patients; diabetic foot ulcers	20	2.5	7 and 14 days after the first HBOT-session	Catalase, MDA, SOD	Catalase: increase post-session #14 MDA: increase post-session #14 SOD: increase post-session #14	
Matzi et al. [82]	2015	In vivo	23 healthy volunteers	1	2.2	During HBOT and immediately post-HBOT	8-hydroxy-deoxyguanosine, GPx, MDA, oxidized low-density lipoprotein, plasma carbonyl proteins	8-hydroxydeoxyguanosine: decrease GPx: increase during HBOT MDA: increase during HBOT Plasma carbonyl proteins: decrease during HBOT *Oxidized low-density lipoprotein: no significant differences were found*	
Morabito et al. [43]	2011	In vivo	6 healthy male, well-trained recreational divers	1	1.6 (dive)/2.2 (dive)	Immediately post-dive	Catalase, hydrogen peroxide, intracellular calcium concentration	Catalase: increase in 2.2 ATA group Hydrogen peroxide: decrease in 2.2 ATA group Intracellular calcium concentration: decrease	No significant differences in catalase and hydrogen peroxide levels were found in the 1.6 ATA group
Muth et al. [76]	2004	In vivo	17 healthy male volunteers	1	2.5	Immediately post-HBOT	Catalase, glutathione, glutathione disulfide, GPx, isoprostanes, MDA, SOD, tail moment	Isoprostanes: increase *Catalase, glutathione, glutathione disulfide, GPx, MDA, SOD, tail moment: no significant differences were found*	
Niu et al. [71]	2013	In vitro	Disc tissue from degenerated lumbar intervertebral discs	3	2.5	24 h after each session	Nitric oxygen	Decrease post-session #2 and post-session #3	
Niu et al. [52]	2019	In vitro	Disc tissues from degenerated lumbar intervertebral discs	3	2.5	12 h post-HBOT	Inducible NOS	Decrease	
Niu et al. [72]	2011	In vitro	Disc tissue from degenerated lumbar intervertebral discs	3	2.5	24 h after each session	Nitric oxygen	Decrease post-session #2 and post-session #3	
Paprocki et al. [89]	2020	In vivo	23 patients; difficult-to heal skin wounds following mechanical injuries	25	2.5	Post-session #1 and #25	Catalase, GPx, SOD, TBARS	*No significant differences were found*	
Paprocki et al. [85]	2019	In vivo	40 patients; sudden sensorineural hearing loss	14	2.5	5 min post-session #1 and post-session #14	Catalase, GPx, SOD, TBARS	Catalase: decrease post-session #1 GPx: increase post-session #14 SOD: decrease post-session #14 TBARS: increase in the erythrocytes post-session #14	No significant differences in TBARS levels in the plasma were found
Puthucheary et al. [69]	2006	In vivo	15 patients	1	2.4	Immediately post-HBOT	(exhaled) Nitric oxygen	Decrease	
Resanovic et al. [53]	2019	In vivo	19 patients; type 1 diabetes mellitus	10	2.4	?	Free fatty acid, inducible NOS, nitrate/nitrite	Free fatty acid: decrease Inducible NOS: decrease Nitrate/nitrite: decrease	
Rocco et al. [87]	2001	In vivo	15 healthy volunteers	1	2/2.8	During HBOT and 30 min post-HBOT	TBARS	Increase	Only pressurization (without breathing 100% O2) has approximately the same effect
Rockswold et al. [79]	2010	In vivo	69 patients; severe traumatic brain injury	1	1.5	?	Isoprostanes	*No significant differences were found*	
Rossignol et al. [101]	2007	In vivo	18 patients; children with autism	40	1.3/1.5	Within 24 h post-HBOT	Glutathione disulfideSSG	*No significant differences were found*	
Rothfuss et al. [95]	2001	In vitro	Human lymphocytes	1	3	1 h, 4 h, 8 h, 12 h, and 24 h post-HBOT	Heme oxygenase-1, tail moment	Heme oxygenase-1: increase as of 4 h post-HBOT Tail moment: increase	
Rothfuss et al. [96]	2002	In vitro	Human lymphocytes	1	1.5	1 h, 4 h, 8 h, 12 h, and 24 h post-HBOT	Heme oxygenase-1, tail moment	Heme oxygenase-1: increase as of 4 h post-HBOT Tail moment: increase	
Shaw et al. [66]	2009	In vitro	Human platelets	1	2.2	?	Nitrate, nitrite	*No significant differences were found*	The level of significance was not determined
Shyu et al. [40]	2008	In vitro	Bone marrow-derived human mesenchymal stem cells	1	2.5	?	ROS	Increase	
Sinan et al. [165]	2016	In vivo	33 patients; various disorders	20	2.4	Post-session #1 and #20	SOD	*No significant differences were found*	
Speit et al. [97]	2000	In vivo	14 healthy volunteers	1	2.5	Immediately or 1 day post-HBOT	catalase, heme oxygenase-1, SOD, tail moment	Heme oxygenase-1: increase *Catalase, SOD, tail moment: no significant differences were found*	
Sureda et al. [49]	2014	In vivo	9 mail professional divers	1	6 (dive)	30 min and 3 h post-dive	Inducible NOS, MDA, nitrite, nitrotyrosine, nitric oxide	Nitrite: increase 3 h post-dive Nitrotyrosine: increase Nitric oxide: increase *Inducible NOS, MDA: no significant differences were found*	
Sureda et al. [62]	2016	In vivo	14 patients; chronic non-healing wound	20	2.2	Pre- and 2 h post-session #1, #5, and #20	Catalase, glutathione reductase, GPx, MDA, myeloperoxidase, nitrite, SOD	Catalase: increase post-session #1 and post-session #5 in plasma MDA: decrease pre-session #20 and post-session #20 Myeloperoxidase: decrease post-session #1, post-session #5, and post-session #20 *Glutathione reductase, GPx, nitrite, SOD: no significant differences were found*	No significant differences were found in catalase levels in erythrocytes
Sureda et al. [55]	2009	In vivo	7 male preprofessional divers	1	5 (dive)	Immediately and 3 h post-dive	Catalase, glutathione reductase, GPx, MDA, nitrite, protein carbonyl derivates, SOD	Catalase: increase immediately post-dive in plasma Nitrite: increase 3 h post-dive SOD: increase 3 h post-dive in plasma *Glutathione reductase, GPx, MDA, protein carbonyl derivates: no significant differences were found*	No significant differences were found in catalase and SOD levels in erythrocytes
Taraldsoy et al. [70]	2007	In vivo	8 patients; chronic radiation-induced injury	20	2.3	Post-session #1 and #19	(exhaled) Nitric oxide	Decrease	
Taylor et al. [90]	2012	In vivo	6 healthy, recreationally active, non-smoking male volunteers	1	2.8	Within 1 h post-HBOT and 5 h post-HBOT	TBARS	*No significant differences were found*	
Teksam et al. [83]	2019	In vivo	54 patients; children with CO poisoning	1	5	Within 1 h post-HBOT	8-hydroxy-deoxyguanosine, catalase, glutathione, GPx, MDA, plasma carbonyl proteins, SOD	*No significant differences were found*	
Tepic et al. [33]	2018	In vivo	50 patients; type 2 diabetes mellitus	10	1.7	Post-session #3, #5, #7, and #10	Catalase, hydrogen peroxide, nitrite, SOD, superoxide-ion, TBARS	Catalase: increase post-session #3 in group without vascular complications and post-session #10 in group with vascular complications Hydrogen peroxide: decrease post-session #3 in group without vascular complications Nitrite: increase post-session #3 in group with vascular complications Superoxide-ion: increase post-session #3 and post-session #10 in group with vascular complications *SOD, TBARS: no significant differences were found*	
Thom et al. [47]	2011	In vivo	8 patients; diabetes mellitus	20	2	?	NOS	Increase	
Tillmans et al. [98]	2019	In vitro	Peripheral blood mononuclear cells from 49 healthy male subjects	1	4	Immediately post-HBOT and 18 h post-HBOT	Tail moment	*No significant differences were found*	
Uusijärvi et al. [61]	2015	In vivo	19 healthy volunteers	1	2.5	During HBOT, 5 min post-HBOT and 30 min post-HBOT	Nitrate, nitrite, nitric oxygen	Nitrite: decrease during HBOT and 5 min post-HBOT NO: decrease in exhaled values *Nitrate: no significant differences were found*	No significant differences were found in the NO values in the plasma
Wang et al. [50]	2011	In vivo	77 patients; diabetic foot ulcers	20	2.5	?	Endothelial NOS	*No significant differences were found*	
Wang et al. [51]	2009	In vivo	74 patients; diabetic foot ulcers	30	2.5	?	Endothelial NOS	*No significant differences were found*	
Wang et al. [73]	2011	In vitro	Disc tissue from lumbar intervertebral discs	3	2.5	24 h post-HBOT	Nitric oxygen	Decrease	
Wang et al. [48]	2020	In vivo	78 patients; spinal cord injury	30	2	?	Endothelial NOS	Increase	
Yuan et al. [54]	2014	In vitro	Atricular cartilage specimens	1	2.5	24 h post-HBOT	Inducible NOS	Decrease	
Zhou et al. [41]	2018	In vitro	Human umbilical vein endothelial cells	1	2.8	?	ROS	Increase	

^a^ All studies used a dry exposure in a hyperbaric chamber, unless ‘dive’ is specified. ^b^ In minutes (min), hours (h), days, weeks, or months post-HBOT. The baseline measurement point has not been included. ? No information on the moment of sample taking (or just ‘post-HBOT’) was noted in the study.

**Table A4:** Retrieved results on inflammation markers in detail.

Study	Year	Design	Subjects	Methods			Type Inflammation Marker	Outcome	Remarks
				Amount of Sessions	Maximum Pressure (ATA) ^a^	Moment of Sample Taking ^b^			
Akcali et al. [104]	2018	In vivo	40 patients; CO poisoning	1	2.4	6 h post-HBOT	hs-CRP, IL-6, IL-10	*No significant differences were found*	
Alex et al. [127]	2005	In vivo	64 patients; on-pump coronary artery bypass grafting	3 (within 24 h)	1.5/2.4	Preoperative (4 h post-HBOT), 2 h postoperative, and 24 h postoperative	IL-6, IL-8, TNF-α	IL-8: decrease preoperative TNF-α: increase 2 h postoperative *IL-6: no significant differences were found*	On-pump coronary artery bypass grafting in the follow-up period
Anguiano-Hernandez et al. [125]	2019	In vivo	18 patients; diabetic foot ulcers	20	1.4	Post-session #20	IFN-γ, IL-4, IL-6, IL-10, NF-κB	IL-6: increase NF-κB: decrease in the nucleus *IFN-γ, IL-4, IL-10: no significant differences were found*	No significant differences in NF-κB levels were seen in the cytosol. The levels of IL-4 were below detection limits.
Aydin et al. [113]	2013	In vivo	48 patients; diabetic foot ulcers	30	2.4	?	Insulin-like growth factor-1	Increase	The level of significance was not determined
Baiula et al. [120]	2020	In vivo	30 patients; chronic non-healing wound	15	2.4	Immediately post-session #4, #8, #12, and #15 and 1 month post-HBOT	IL-1β, TNF-α	IL-1β: decrease as of post-session #12 TNF-α: decrease as of post-session #12	
Benson et al. [121]	2003	In vitro	Peripheral blood mononuclear cells	1	2.4	?	IL-1β, TNF-α	IL-1β: decrease TNF-α: decrease	LPS-, lipid A- and PHA-induced IL-1β and TNF-α production was measured
Bent et al. [112]	2012	In vivo	10 children; autism spectrum disorder	80	1.5	Post-session #40 and #80	Granulocyte-colony stimulating factor, IFN-α, IFN-γ, IL-1α, IL-1β, IL-2, IL-3, IL-4, IL-5, IL-6, IL-7, IL-8, IL-10, IL-12(p40), IL-12(p70), IL-13, IL-15, IL-17, TNF-α	*No significant differences were found*	
Bosco et al. [4]	2014	In vivo	21 patients; pancreactico-duodenectomy	1	2.5	Post-HBOT, 1 day postoperative, and 7 days postoperative	IL-1, IL-6, IL-8, IL-10, IL-12p70, TNF-α	IL-6: decrease IL-10: decrease *IL-1, IL-8, IL-12p70, TNF-α: no significant differences were found*	Pancreaticoduodenectomy in the follow-up period
Bosco et al. [35]	2018	In vivo	23 patients; unilateral femoral head necrosis	60	2.5	Post-session #15, #30, and #60 and pre-session #31	IL-1β, IL-6, TNF-α	IL-6: decrease TNF-α: decrease *IL-1β: no significant differences were found*	
Chang et al. [102]	2020	In vivo	10 healthy male volunteers	1	2.8	30 min, 2 days, and 1 week post-HBOT	Albumin	*No significant differences were found*	
Chen et al. [106]	2017	In vivo	38 patients; diabetic foot ulcers	20	2.5	Post-session #10, post-session #20, and 2 weeks post-HBOT	CRP, erythrocyte sedimentation rate	CRP: decrease 2 weeks post-session Erythrocyte sedimentation rate: decrease 2 weeks post-session	
Chen et al. [88]	2018	In vivo	50 patients; acute non-cardioembolic stroke	1	2.5	1 month post-HBOT	hs-CRP	*No significant differences were found*	
Chen et al. [67]	2007	In vivo	61 patients; diabetes mellitus type 2	3	2.5	Immediately post-session #1 and #3	Insulin-like growth factor-1, IL-8	*No significant differences were found*	
Chong et al. [118]	2013	In vivo	17 patients; thermal burns	2	2.4	?	IFN-γ, IL-1β, IL-2, IL-4, IL-5, IL-6, IL-10, IL-12(p70), IL-13, TNF-α	IFN-γ: increase *IL-1β, IL-2, IL-4, IL-5, IL-6, IL-10, IL-12(p70), IL-13: no significant differences were found*	Outcome on TNF-α is not reported
Dhamodharan et al. [45]	2019	In vivo	37 patients; diabetic foot ulcer	25	2.2	20 days after the first HBOT-session	CRP, IFN-γ, IL-1α, IL-8, IL-10, TNF-α	CRP: decrease IFN-γ: decrease IL-1α: increase IL-8: increase IL-10: increase *TNF-α: no significant differences were found*	
Fan et al. [122]	2020	In vivo	122 patients; Parkinson’s disease dementia	40	20 MPA	?	IL-1β, IL-6	IL-1β: decrease IL-6: decrease	
Fildissis et al. [128]	2004	In vitro	Blood samples from 16 healthy volunteers	1	2.4	?	IL-6, IL-8, TNF-α	*No significant differences were found*	
Guggino et al. [133]	2019	In vivo	36 patients; primary fibromyalgia	40	2	1 month post-HBOT	IFN-γ, IL-9, IL-17, IL-22, TNF-α	IFN-γ: decrease TNF-α: decrease *IL-9, IL-17, IL-22: no significant differences were found*	
Hao et al. [111]	2020	In vivo	30 patients; plastic surgery	7	2	24 h post-HBOT	Granulocyte-colony stimulating factor, IFN-α2, IFN-γ, IL-1α, IL-1β, IL-1Ra, IL-2, IL-3, IL-4, IL-5, IL-6, IL-7, IL-8, IL-9, IL-10, IL-12p40, IL-12p70, IL-13, IL-15, IL-17, TNF-α	IL-1Ra: increase IL-12p40: decrease *Granulocyte-colony stimulating factor, IFN-α2, IFN-γ, IL-1α, IL-1β, IL-2, IL-3, IL-4, IL-5, IL-6, IL-7, IL-8, IL-9, IL-10, IL-12p70, IL-13, IL-15, IL-17, TNF-α: no significant differences were found*	The samples were taken during surgery
Hedetoft et al. [114]	2020	In vivo	39 patients; diabetes	30	2.4	Post-session #30 and after 90 days follow-up	Insuline-like growth factor-1	Decrease after 90 days follow-up in the diabetic group	No significant differences in IGF-1 values were seen in the non-diabetic group
Hou et al. [129]	2019	In vivo	132 patients; brain tumor	10	1.8	?	IL-6, TNF-α	IL-6: decrease TNF-α: decrease	
Irawan et al. [116]	2018	In vivo	36 patients; diabetic foot ulcers	20	2.4	Post-session #20	Albumin	Increase	
Irawan et al. [117]	2018	In vivo	30 patients; diabetic foot ulcers	10	2.4	?	Albumin	*No differences were found*	The level of significance was not determined
Karadurmus et al. [103]	2010	In vivo	28 patients; diabetic foot ulcers	30	2.4	Post-session #10, #20, and #30	hs-CRP	Decrease	
Kendall et al. [46]	2012	In vitro	Human umbilical vein endothelial cells	1	2.4	5 h and 22.5 h post-HBOT	IL-8	Decrease 5 h post-HBOT	
Li et al. [84]	2017	In vivo	78 patients; chronic diabetic wounds	By average 48	2.4	30 days after the first session	CRP, TNF-α	CRP: decrease TNF-α: decrease	
Li et al. [58]	2018	In vivo	115 patients; coronary artery disease with drug-eluting stents	24	2	?	hs-CRP	Decrease	
Li et al. [59]	2019	In vivo	115 patients; coronary artery disease with coronary stent implantation	24	2	?	hs-CRP	Decrease	
Li et al. [60]	2018	In vivo	98 patients; slow coronary flow	24	2	?	hs-CRP	Decrease	
Lin et al. [130]	2018	In vivo	57 patients; peripheral arterial occlusive disease	10–15	2.5	Post-session #3 and #5	IL-6	Decrease	
Liu et al. [137]	2020	In vivo	140 patients; unilateral idiopathic sudden sensorineural hearing loss	15	2	?	NF-κB, TNF-α	NF-κB: decrease TNF-α: decrease	
MacKenzie et al. [126]	2000	In vitro	Peripheral blood mononuclear cells	1 (duration: 48 h)	1.7	7 days post-HBOT	IFN-γ, IL-4, IL-10	*No significant differences were found*	The IL-4 values were below detection limits
Madden et al. [139]	2011	In vivo	10 healthy male volunteers	1	2.8	Immediately post-HBOT and 4 h post-HBOT	NF-κB	Increase 4 h post-HBOT	
Nasole et al. [135]	2014	In vivo	27 patients; chronic leg wounds	40	2.5	7 and 14 days after the first HBOT-session	TNF-α	*No significant differences were found*	
Niu et al. [71]	2013	In vitro	Disc tissue from degenerated lumbar intervertebral discs	3	2.5	24 h after each session	IL-1β	Decrease	
Niu et al. [52]	2019	In vitro	Abnormal disc tissues from degenerated lumbar intervertebral discs	3	2.5	1 h post-session #3	NF-κB	Decrease in the nucleus	
Niu et al. [72]	2001	In vitro	Disc tissue from degenerated lumbar intervertebral discs	3	2.5	24 h after each session	IL-1β	Decrease	
Resanovic et al. [53]	2019	In vivo	19 patients; type 1 diabetes mellitus	10	2.4	?	CRP, NF-κB	CRP: decrease NF-κB: decrease	
Rocco et al. [87]	2001	In vivo	15 healthy volunteers	1	2.8	During HBOT and 30 min post-HBOT	IL-1β, IL-6, TNF-α	TNF-α: increase during HBOT *IL-1β, IL-6: no significant differences were found*	
Romero-valdovinos et al. [115]	2011	In vitro	Dermal fibroblasts	30	3	?	Insulin-like growth factor-1	Decrease in the keloid fibroblast group	The nonkeloid fibroblasts did not express IGF-1
Rosario et al. [131]	2018	In vivo	6 patients; ischemic stroke	40	2	?	IL-6, TNF-α	IL-6: decrease TNF-α: decrease	
Rossignol et al. [101]	2007	In vivo	18 patients; children with autism	40	1.3/1.5	Within 24 h post-HBOT	CRP	Decrease	In the group with lower CRP baseline levels no decrease in CRP concentrations was seen
Rothfuss et al. [96]	2002	In vitro	Human lymphocytes	2	1.5/3	4 h, 8 h, and 24 h post-HBOT	Ferritin	*No significant differences were found*	
Schnittger et al. [110]	2004	In vivo	8 patients; CO poisoning	3 (within 24 h)	2.8	Before and after each session	Granulocyte-colony stimulating factor, IL-8	Granulocyte-colony stimulating factor: increase pre- and post-session #2 in the patient group *IL-8: no significant differences were found*	No significant differences in granulocyte-colony stimulating factor values were seen in the control group
Semadi et al. [138]	2019	In vivo	32 patients; diabetic foot ulcer	20	2.4	Post-session #20	TNF-α	Decrease	
Song et al. [132]	2018	In vivo	134 patients; keloid surgery and radiotherapy	14	2	Post-operation #2	IL-6, NF-κB, TNF-α	IL-6: decrease NF-κB: decrease TNF-α: decrease	Keloid surgery and radiotherapy in the follow-up period
Sun et al. [119]	2018	In vivo	80 patients; brain injury	20	2	8 h, 24 h, 48 h, and 72 h post-HBOT	IL-1β, TNF-α	TNF-α: decrease *IL-1β: no significant differences were found*	Baseline values of TNF-α were significantly higher in the HBOT-group compared to the control group
Sun et al. [140]	2019	In vivo	79 patients; acute spinal cord injury	30	2	Post session #1, #3, #7, #10, and #30	NF-κB	Decrease post-session #3, #7, #10, and #30	
Sureda et al. [62]	2016	In vivo	14 patients; chronic non-healing wound	20	2.2	Pre- and 2 h post-session #1, #5, and #20	IL-6	Increase 2 h post-session #1, #5, and #20	
Top et al. [107]	2007	In vivo	38 patients; type 2 diabetes mellitus	?	2	2 weeks after the first HBOT-session	CRP, erythrocyte sedimentation rate	CRP: decrease Erythrocyte sedimentation rate: increase	
Vezzani et al. [105]	2016	In vivo	30 patients; CO poisoning	1	2.5/2.8	Immediately post-HBOT	CRP, IL-6, IL-8, IL-10, TNF-α	IL-8: decrease in the control group IL-10: decrease in the patient group TNF-α: decrease *CRP, IL-6: no significant differences were found*	No significant differences regarding IL-8 and IL-10 were seen in respectively the patient group and the control group
Wang et al. [134]	2011	In vitro	Human coronary artery endothelial cells	1	2.5	?	TNF-α	Increase during HBOT	The TNF-α values returned to the baseline values before the HBOT ended
Wang et al. [73]	2011	In vitro	Disc tissue from lumbar intervertebral discs	1	2.5	48 h, 96 h, and 144 h post-HBOT	IL-1β	Decrease	
Weisz et al. [124]	1997	In vivo	7 patients; perianal Crohn’s disease	20/40	2.5	Immediately post-session #1, 24 h post-session #1, 20 h post-session #2, and 20 h post-session #20	IL-1, IL-6, TNF-α	IL-1: decrease immediately and 24 h post-session #1 and 20 h post-session #2 IL-6: decrease immediately and 24 h post-session #1 and 20 h post-session #2 TNF-α: decrease immediately post-session #1 and 20 h post-session #2	
Wilkinson et al. [123]	2015	In vivo	19 male volunteers; overweight/obese	4	2	During HBOT, immediately post-session #4, and 24 h post-session #4	IL-1Ra, IL-6, IL-18, TNF-α	IL-6: increase during HBOT and immediately post-session #4 in the non-diabetes group TNF-α: decrease 24 h post-session #4 *IL-1Ra, IL-18: no significant differences were found*	
Xie et al. [109]	2007	In vivo	60 patients; craniocerebral injury	10	2.5	Within 24 h post-HBOT	CRP	Decrease	
Yildiz et al. [108]	2016	In vivo	43 patients; hidradenitis suppurativa	20	2.4	Post-session #20 and 6 weeks post-HBOT	CRP, erythrocyte sedimentation rate	CRP: decrease Erythrocyte sedimentation rate: decrease	
Yoshinoya et al. [136]	2020	In vitro	Adipose-derived stem cells	5	2/3	90 min before and immediately after each session	TNF-α	*No significant differences were found*	The TNF-α values were below detection limits

^a^ All studies used a hyperbaric chamber for pressurization. ^b^ In minutes (min), hours (h), days, weeks, or months post-HBOT. The baseline measurement point has not been included. ? No information on the moment of sample taking (or just ‘post-HBOT’) was noted in the study.

**Table A5:** Retrieved results on angiogenesis markers in detail.

Study	Year	Design	Subjects	Methods			Type Angiogenesis Marker	Outcomes	Remarks
				Amount of Sessions	Maximum Pressure (ATA) ^a^	Moment of Sample Taking ^b^			
Anguiano-Hernandez et al. [125]	2019	In vivo	18 patients; diabetic foot ulcers	20	1.4	Post-session #20	HIF-1α, insulin-like growth factor binding protein-3, VEGF	Insulin-like growth factor binding protein-3: increase in nucleus and fibroblast VEGF: increase in the cytosol *HIF-1α: no significant differences were found*	No significant differences in insulin-like growth factor binding protein-3 and VEGF levels were found in the cytoplasm and nucleus, respectively
Bent et al. [112]	2012	In vivo	10 children; autism spectrum disorder	80	1.5	Post-session #40 and #80	Granulocyte-macrophage colony-stimulating factor, TGF-β1, TGF-β2	*No significant differences were found*	
Chang et al. [102]	2020	In vivo	10 healthy male volunteers	1	2.8	30 min, 2 days, and 1 week post-HBOT	FGF21	*No significant differences were found*	
Chen et al. [67]	2007	In vivo	61 patients; diabetes mellitus type 2	3	2.5	Immediately post-session #1 and #3	Insulin-like growth factor-2, insulin-like growth factor binding protein-1, insulin-like growth factor binding protein-3	Insulin-like growth factor binding protein-1: decrease post-session #1 and (less prominent) post-session #3 *Insulin-like growth factor-2, insulin-like growth factor binding protein-3: no differences were found*	No significance levels were determined
Cheung et al. [37]	2018	In vitro	Umbilical cord blood enriched with CD34-cells	1	2.5	24 h post-HBOT	Erythropoietin-receptor	*No significant differences were found*	
Chong et al. [118]	2013	In vivo	17 patients; thermal burns	2	2.4	?	VEGF	*No significant differences were found*	
Dhamodharan et al. [45]	2019	In vivo	37 patients; diabetic foot ulcer	25	2.2	20 days after the first HBOT-session	EGF, FGF-2, platelet-derived growth factor, VEGF	EGF: increase FGF-2: increase VEGF: increase *Platelet-derived growth factor: no significant differences were found*	
Grimberg-Peters et al. [44]	2016	In vitro	Neutrophils from severely injured patients and healthy volunteers	1	2	?	ERK, p38 MAPK	p38 MAPK: decrease *ERK: no significant differences were found*	The decrease in p38 MAPK levels was only found after 3h of stimulation with PMA
Hao et al. [111]	2020	In vivo	30 patients; plastic surgery	7	2	24 h post-HBOT	EGF, FGF-2, granulocyte-macrophage colony-stimulating factor, platelet-derived growth factor-AA, platelet-derived growth factor-BB, TGF-α, VEGF	Platelet-derived growth factor-BB: decrease *EGF, FGF-2, granulocyte-macrophage colony-stimulating factor, platelet-derived growth factor-AA, TGF-α, VEGF: no significant differences were found*	The samples were taken during surgery.
Jung et al. [143]	2010	In vivo	86 patients; acute hearing loss/tinnitus	10	1.55	1, 2, 5, and 10 days after the first HBOT-session	bFGF, VEGF	bFGF: decrease *VEGF: no significant differences were found*	
Kang et al. [147]	2004	In vitro	Fibroblast primary cell lines	7	1.5/2/2.5/3	1 day, 3 days, 5 days, and 7 days after the first HBOT-session	bFGF, TGF-β1, VEGF	*No significant differences were found*	Administration of 100% oxygen significantly increased the bFGF levels at day 1, yet pressurization had no extra effect. The TGF-β1 values were below detection limits.
Kunnavatana et al. [148]	2005	In vitro	Fibroblast cell line	7	2	1 day, 3 days, 5 days, and 7 days after the first HBOT-session	bFGF, TGF-β1, VEGF	*No significant differences were found*	
Lee et al. [142]	2006	In vitro	Human umbilical vein endothelial cells	1	2.5		ERK, VEGF	ERK: increase VEGF: increase	
Li et al. [84]	2017	In vivo	78 patients; chronic diabetic wounds	By average 48	2.4	30 days after the first HBOT-session	Ang-2, VEGF	Ang-2: increase VEGF: increase	
Lin et al. [130]	2018	In vivo	57 patients; peripheral arterial occlusive disease	10 of 15	2.5	Post-session #3 and #5	Hematopoietic growth factor, HIF-1α, stromal cell-derived factor-1α, VEGF	Hematopoietic growth factor: increase HIF-1α: decrease Stromal cell-derived factor-1α: increase VEGF: increase	
Lin et al. [144]	2002	In vitro	Human umbilical vein endothelial cells	1	2.5	?	Ang-1, Ang-2, Tie-2, VEGF	*No significant differences were found*	Administration of 100% oxygen significantly increased the Ang-2 levels, yet pressurization had no extra effect
Mutzbauer et al. [145]	2015	In vivo	16 divers	3	1.4 (dive)	Within 1 h pre- and post-dive	Erythropoietin	Decrease post-dive #2 and #3	
Nasole et al. [135]	2014	In vivo	27 patients; chronic leg wounds	40	2.5	7 and 14 days after the first HBOT-session	EGF, VEGF	*No significant differences were found*	
Niu et al. [71]	2013	In vitro	Disc tissue from degenerated lumbar intervertebral discs	3	2.5	30 min and 60 min post-session #3	ERK1/2, p38 MAPK	ERK1/2: decrease p38 MAPK: decrease	Phosphorylation of p38 MAPK and ERK has been measured
Niu et al. [52]	2019	In vitro	Abnormal disc tissues from degenerated lumbar intervertebral discs	3	2.5	ERK1/2, p38 MAPK: 15 min and 30 min post-session #3 MMP-3, MPP-9, MMP-13: 12 h after each session	ERK1/2, MMP-3, MMP-9, MMP-13, p38 MAPK	ERK1/2: decrease 30 min post-session #3 MMP-3: decrease post-session #2 and #3 MMP-9: decrease post-session #2 and #3 MMP-13: decrease post-session #2 and #3 p38 MAPK: decrease	Phosphorylation of p38 MAPK and ERK has been measured
Niu et al. [72]	2011	In vitro	Disc tissue from degenerated lumbar intervertebral discs	3	2.5	MMP-3: 24 h after each session p38 MAPK: 15 min, 30 min, and 60 min post-session #3	MMP-3, p38 MAPK	MMP-3: decrease post-session #3 p38 MAPK: decrease	Phosphorylation of p38 MAPK and ERK has been measured
Resanovic et al. [53]	2019	In vivo	19 patients; type 1 diabetes mellitus	10	2.4	?	Akt, ERK1/2	Akt: decrease ERK1/2: decrease	
Romero-valdovinos et al. [115]	2011	In vitro	Dermal fibroblasts	30	3	?	TGF-β	TGF-β: decrease	
Semadi et al. [138]	2019	In vivo	32 patients; diabetic foot ulcer	20	2.4	Post-session #20	VEGF	Increase	
Shyu et al. [40]	2008	In vitro	Bone marrow-derived human mesenchymal stem cells	1	2.5	1 h, 2 h, 4 h, and 6 h post-HBOT	PGF	Increase	The increase in PGF levels was higher at 1h and 2h post-HBOT compared to 4h and 6h post-HBOT
Song et al. [132]	2018	In vivo	134 patients; keloid surgery and radiotherapy	14	2	Post-operation #2	HIF-1α, VEGF	HIF-1α: decrease VEGF: decrease	Keloid surgery and radiotherapy in the follow-up period
Sureda et al. [62]	2016	In vivo	14 patients; chronic non-healing wound	20	2.2	Pre- and 2 h post-session #1, #5, and #20	VEGF	Increase post-session #1, post-session #5, and post-session #20	
Thom et al. [47]	2011	In vivo	8 patients; diabetes mellitus	20	2	Pre- and post-session #1, #10, and #20	HIF-1α	Decrease post-session #1, #10, and #20	No significant differences in HIF-1α levels were found pre-session
Tra et al. [141]	2014	In vitro	Tissue-engineered mucosa and human umbilical vein endothelial cells	1/3/5	2.4	Immediately post-HBOT	bFGF, hematopoietic growth factor, keratinocyte growth factor, PGF, VEGF	bFGF: an increase in the one-session group and a decrease in the three- and five-session group Hematopoietic growth factor: increase in the one-session group Keratinocyte growth factor: increase in the one- and five-session group PGF: an increase in the one- and five-session group and a decrease in the three-session group VEGF: increase in the five-session group	
Wang et al. [50]	2011	In vivo	77 patients; diabetic foot ulcers	20	2.5	?	EGF, VEGF	*No significant differences were found*	
Wang et al. [51]	2009	In vivo	74 patients; diabetic foot ulcers	30	2.5	?	VEGF	*No significant differences were found*	
Wang et al. [48]	2020	In vivo	78 patients; spinal cord injury	30	2	?	Akt, PI3K	Akt: increase PI3K: increase	
Yoshinoya et al. [136]	2020	In vitro	Adipose-derived stem cells	5	2/3	90 min before and immediately after each session	EGF, hematopoietic growth factor, TGF-β	TGF-β: decrease post-session #3 in the 2 ATA group and post-session #4 in the 3 ATA group *EGF, hematopoietic growth factor: no significant differences were found*	The EGF values were below detection limits
Yuan et al. [54]	2014	In vitro	Atricular cartilage specimens	1	2.5	24 h post-HBOT	MMP-3	Decrease	

^a^ All studies used a dry exposure in a hyperbaric chamber, unless ‘dive’ is specified. ^b^ In minutes (min), hours (h), days, or months post-HBOT. The baseline measurement point has not been included. ? No information on the moment of sample taking (or just ‘post-HBOT’) was noted in the study.
